# Resilience as a Mediator of the Association Between Perceived Stigma and Quality of Life Among People With Inflammatory Bowel Disease

**DOI:** 10.3389/fpsyt.2021.709295

**Published:** 2021-08-06

**Authors:** Dan Luo, Meijing Zhou, Lifu Sun, Zheng Lin, Qiugui Bian, Meihong Liu, Shurong Ren

**Affiliations:** ^1^School of Nursing, Nanjing University of Chinese Medicine, Nanjing, China; ^2^Department of Nursing, The First Affiliated Hospital of Nanjing Medical University, Nanjing, China; ^3^School of Clinical Medicine, Nanjing Medical University, Nanjing, China; ^4^School of Nursing, Nanjing Medical University, Nanjing, China; ^5^Department of Gastroenterology, The First Affiliated Hospital of Nanjing Medical University, Nanjing, China

**Keywords:** resilience, perceived stigma, quality of life, mediating effect, inflammatory bowel disease

## Abstract

**Background:** Improving Quality of Life (QOL) is an essential objective in the management of inflammatory bowel disease. An accumulating body of research has been conducted to explore the association between perceived stigma and QOL among patients with chronic illness. Still, underlying mechanisms behind this pathway have not been thoroughly examined.

**Objective:** To investigate (a) the effect of perceived stigma on QOL among patients with inflammatory bowel disease; and (b) the mediating role of resilience in the association between perceived stigma and QOL.

**Methods:** This cross-sectional study included a convenient sample of patients diagnosed with inflammatory bowel disease from four tertiary hospitals in Jiangsu Province, China. Patients completed the Perceived Stigma Scale in Inflammatory Bowel Disease (PSS-IBD), the Resilience Scale for Patients with Inflammatory Bowel Disease (RS-IBD), and the Inflammatory Bowel Disease Questionnaire (IBDQ). A bootstrapping analysis was implemented using the SPSS macro PROCESS.

**Results:** A total of 311 patients with Cohn's disease and ulcerative colitis participated in this study, and 57.6% were men. The mean disease duration was 3.51 ± 1.04 years. Approximately 40% of the sample exceeded the criterion score for moderate stigma. Patients who perceived moderate or severe stigma reported lower QOL compared with those with mild stigma. After controlling for sociodemographic and clinical variables, we observed that perceived stigma was negatively associated with resilience. Moreover, resilience was found to mediate the relationship between perceived stigma and all aspects of QOL.

**Conclusions:** These findings suggested that QOL of patients with inflammatory bowel disease was associated with perceived stigma and resilience and identified the mediating effects of resilience in the relationship between perceived stigma and QOL. Furthermore, this suggests that integrating intervention techniques to target resilience into the QOL improvement program of individuals with perceived stigma is possible.

## Introduction

Inflammatory bowel disease (IBD), mainly comprising Cohn's disease (CD) and ulcerative colitis (UC), are immune-mediated intestinal inflammatory disorders characterized by early-onset. Recently epidemiological data show that approximately 6.9 million individuals worldwide are living with IBD (1). Moreover, the incidence and prevalence of IBD are still increasing, especially in newly industrialized countries. Patients with IBD suffer from abdominal pain, diarrhea, rectal bleeding, and frequent bowel movements throughout their lifetime repeatedly (2, 3). Treatment for IBD largely depends on pharmacological means, but surgery is often needed when complications occur (4). It is estimated that the overall 10-year risk of surgery after diagnosis is 39.5% for CD and 13.3% for UC (5).

The quality of life (QOL) among patients with IBD is of growing significance since the patient-reported outcomes are recognized as primary endpoints by regulatory agencies (6). QOL is a broad multidimensional concept that involves objective and subjective aspects, focusing on the physical, emotional, mental, and social impact of the disease on patients' lives (7). Traumatic childhood experiences, sensory processing patterns, and chronic disease are recognized risk factors for QOL (8, 9). As an uncommon, chronic disease, IBD seriously impairs the QOL of patients (10–12). A recent meta-analysis performed by Knowles and his colleagues indicated that QOL was significantly lower for those with IBD relative to healthy controls (13). QOL among patients with IBD may be influenced by disease symptoms, treatment regimen, and various psychosocial variables (14). Psychosocial variables could affect the patients' psychological and social functions and alter gastrointestinal physiology by activating the microbiota-brain-gut axis, leading to decreased or improved QOL (15–18). Sweeney and her colleagues reported that depression, fear avoidance, and catastrophizing were risk factors for IBD -related pain, while self-efficacy and mental well-being were protective factors (19). Recently, the perceived stigma has been considered a prominent psychosocial variable that damage QOL (20). Therefore, the understanding of QOL should pay attention to the physical distress caused by IBD and consider perceived stigma and other psychosocial factors.

Perceived stigma is defined as individuals' feelings that other persons hold negative attitudes or negative beliefs about them and their condition (21). Risks of incontinence in public, the changes in body image, negative impacts on sexual life, and burdens to others make patients with IBD susceptible to perceived stigma (22). Taft TH et al. investigated the prevalence of stigma in patients with IBD and reported that 84% of participants had perceived stigma (23). Literature outside the field of IBD has demonstrated that higher levels of perceived stigma are associated with poorer QOL (24–26). While perceived stigma is identified as a specific concern for patients with IBD, only one study to date has proved the negative relationship between perceived stigma and QOL (23).

Moreover, the specific mechanisms by which perceived stigma affects QOL in patients with IBD remain elucidated. As not all IBD patients who perceive stigma have poor QOL, it is plausible to assume that some factors may mediate such a connection (27, 28). Resilience, one of the most mentioned positive psychological resources, is usually defined from three perspectives in chronic disease: traits, outcome, and process. For example, (1) traits reflecting the characteristics of tenacity and flexibility in response to disease-related stress; (2) positive health outcomes in high-risk patients; (3) a dynamic process of successful adaptation when exposed to chronic disease (29). There is a debate concerning how to define resilience, but previous research has identified the role of 'resiliency training programs,' which infers it is modifiable (29). In our study, resilience is a quantifiable and modifiable personal quality that enables individuals to bounce back from IBD-related adversity (30). Resilient patients could cope with their disease adaptively. Neurological evidence revealed that resilience could invoke specific brain structures and neural circuits to help the individual to regulate emotion and adopt adaptive social behavior (17, 18). Resilience was reported to positively predict QOL in adolescents with type 1 diabetes and patients with acute myocardial infarction (31). Among people living with IBD, higher levels of resilience were significantly associated with better QOL (32, 33). Perceived stigma from family members, spouse, friends, employers, and colleagues affects resilience. A qualitative study revealed that stigma is more pronounced in less resilient IBD patients, especially in those lacking support networks (34). Self-esteem, mastery, and optimism, which are essential components of resilience, could be reduced by perceived stigma (35). Hsiung et al. found that mastery mediates the association between perceived stigma and QOL in patients with schizophrenia (36). The above evidence suggested that perceived stigma might impair resilience by causing low self-esteem, mastery, and pessimism, sequentially affecting QOL. However, no studies have analyzed the relationship between perceived stigma, resilience, and QOL among patients with IBD to our knowledge.

The present study aims to explore the impact of perceived stigma on QOL and investigate the mediating effects of resilience in the path from perceived stigma to QOL among patients with IBD. First, we hypothesized that perceived stigma was negatively associated with QOL among patients with IBD. Second, we further supposed that resilience could mediate the link between perceived stigma and QOL in these patients.

## Materials and Methods

### Participants and Procedure

Patients with IBD were recruited from the Digestive Department of four tertiary hospitals in Jiangsu Province, China, using consecutive sampling. Participants were referred by their charge nurse if they were (1) diagnosed with UC or CD according to the current diagnostic criteria (2, 3); age≥18 years; (3) disease duration of more than 6 months; (4) education level of all participants was elementary school or above, able to read and understand the questionnaire. Exclusion criteria included: (1) severe mental illness (such as schizophrenia, bipolar disorder, paranoiac psychosis); (2) malignant tumors or other chronic diseases (such as heart failure, diabetes); (3) combined with other intestines or anorectal disease. The first authors introduced the purpose, procedures, and potential benefits and risks of the study. All the patients were asked to give written informed consent if they were eligible for the study. Next, the patients completed a set of self-reported questionnaires in a quiet room at each clinical site, including demographic (age, gender, education level, marital status, residence, family income) and clinical (disease type, duration) information. Modified Truelove and Witts' Severity Index and Harvey Bradshaw Simple Index were used to measure the disease activity of UC and CD patients, respectively, by their nurse in charge. We used G.Power 3.1 to calculate the sample size. The results showed that the data from 257 patients with UC and CD would give sufficient power (0.90) to detect a small effect size (0.05) on quality of life, explained by 13 independent variables (the perceived stigma, resilience, demographic, and clinical variables), with alpha = 0.05.

**Figure 1:**
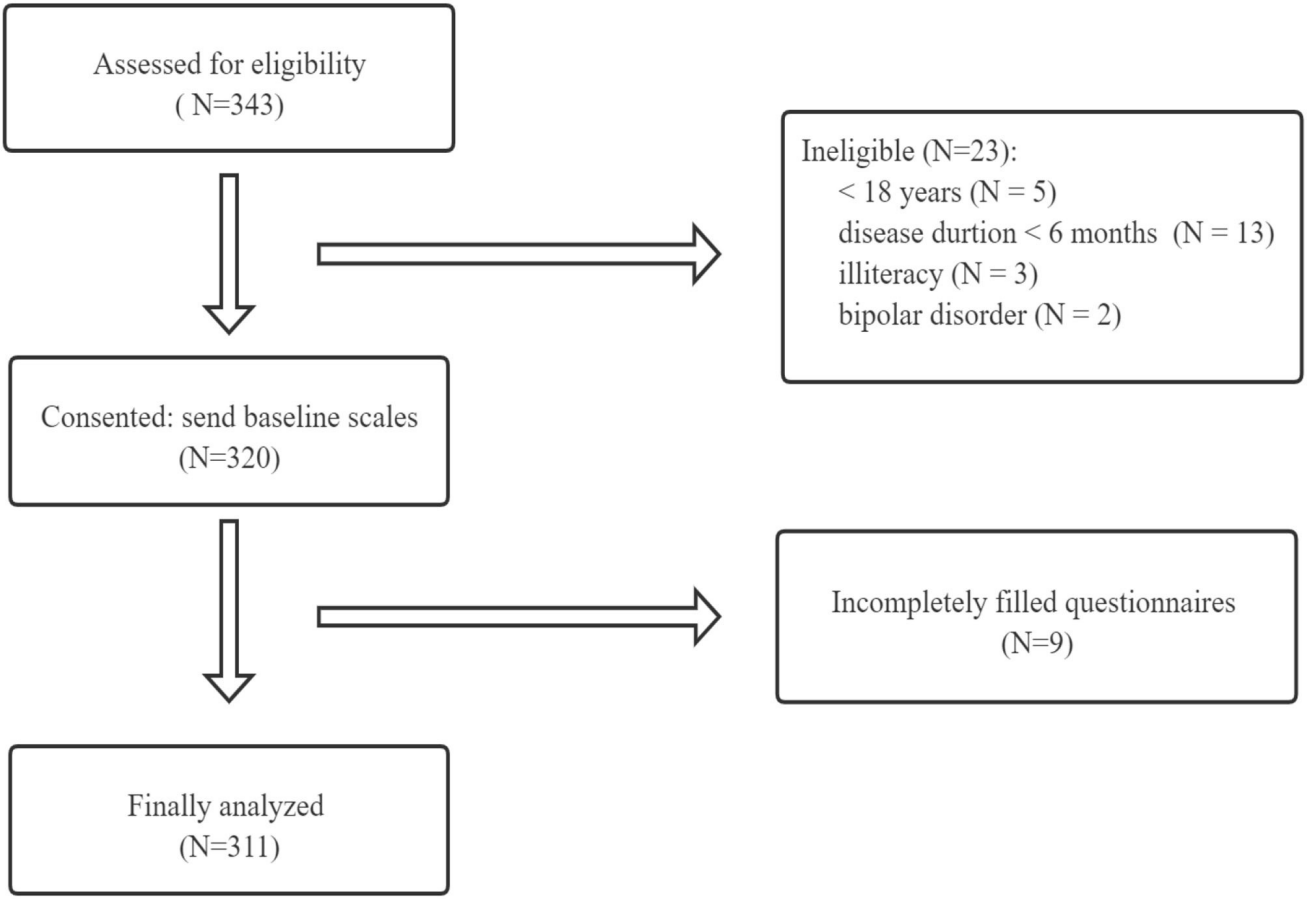
Participant recruitment flow chart.

Of the 343 patients who were interested in participating and screened for eligibility, 320 were eligible. Three hundred twenty patients signed the informed consent and finished the questionnaires. Because of the incompletely filled questionnaires, nine respondents were excluded from the study. Finally, the data of 311 participants were used for the data analysis. Figure 1 showed the participant recruitment process in detail.

### Instruments

#### Perceived Stigma

The Perceived Stigma Scale in IBD (PSS-IBD) is a 10-item instrument designed to determine the presence of perceived stigma (23). Items are rated on a five-point scale ranging from 0 (Never) to 4 (always) and evaluated for six social domains. A total score of perceived stigma can be obtained by summing the scores of all six social dimensions. Scores of 1–80 indicate low stigma, 81–160 represent moderate stigma, and scores equal to or above 161 suggest high stigma. The PSS-IBD has been proved to have adequate reliability in American and Chinese patients with IBD (Cronbach's alpha = 0.89 and 0.96, respectively) (23, 37).

#### Resilience

The Resilience Scale for Patients with Inflammatory Bowel Disease (RS-IBD) is a specific resilience instrument for patients with IBD developed in China (38). The RS-IBD includes 25 items that are classified into six dimensions. Each item is scored on a five-point scale ranging from 1 (not true at all) to 5 (true all the time), with higher scores suggesting higher resilience. The RS-IBD was reported to have adequate reliability and construct validity in Chinese people with IBD (38).

### Quality of Life

Quality of life was assessed using the Inflammatory Bowel Disease Questionnaire (IBDQ), a 32 items instrument developed in Canada (39). The IBDQ comprises four dimensions: bowel symptoms, systemic symptoms, emotional function, and social function. The IBDQ rated on a seven-point Likert scale, ranging from 1 to 7. The scale's total score can be calculated by summing the scores of all dimensions, with higher scores indicating better QOL. The Chinese version of the IBDQ was reported to exhibit adequate internal consistency reliability (Cronbach's alpha, 0.95), split-half reliability (0.90), and construct validity (four factors accounted for 60.99% of the variation) (40).

### Disease Activity

For UC, the disease activity was assessed by modified Truelove and Witts' Severity Index (41, 42). Truelove and Witts' Severity Index involves the number of bloody stools per day, body temperature, pulse, hemoglobin, and erythrocyte sedimentation rate. Patients with UC stratified as having four scores or less were regarded as being in remission, while those with four or more were in the active stage. We adopted the Harvey Bradshaw Simple Index to assess the disease activity of CD. This scale is widely used in clinical practice and scientific research (41, 43). It comprises five aspects: overall well-being, the severity of abdominal pain, the daily number of liquid stools, abdominal lumps, and complications. Patients with CD categorized as having four scores or less were regarded as being in remission, while those with four or more were considered with active disease.

### Statistical Analyses

Statistical analyses were performed using IBM SPSS 20.0 for Windows (IBM Corp., Armonk, NY, USA). In this cross-sectional study, descriptive statistics were computed for all study variables. Before the analysis, the Kolmogorov-Smirnov test was used to determine whether the numeric variables showed a normal distribution. Next, the *t*-test and analysis of variance (ANOVA) were applied to compare the QOL among patients with different sociodemographic and clinical characteristics. The relationships between perceived stigma, resilience, and QOL were assessed utilizing Pearson's bivariate Correlation. Finally, we performed a bias-corrected bootstrapping analysis (with 5000 resamples) using the SPSS macro–PROCESS Model 4 to verify the mediating effects of resilience. The mediating effect was considered statistically significant if the 95% bootstrap confidence interval did not contain zero (44). Sub-group analysis was performed based on disease activity (remission vs. active disease). The interactions of disease activity and perceived stigma and resilience were also explored. All statistical tests were performed at a 0.05 level of significance.

## Results

### Participants' Sociodemographic and Clinical Characteristics

The final sample consisted of 311 patients with IBD. Their mean age was 33.70 ± 11.62 (mean ± standard deviation) years, and 57.6% were men. Nearly half of the participants (42.7%) had a higher education level, and 224 participants (72.0%) lived in the cities. Seventy-nine patients (25.4%) were divorced, and 149 patients (47.9%) were married. More than half of the sample (60.1%) had an annual household income of 50,000 Yuan or more. On disease type, 69.5% of patients were diagnosed with CD. The mean duration time since diagnosis was 3.51 ± 1.04 years. Most of them (67.2%) were in remission (Table 1).

**Table 1:** Demographic and clinical characteristic differences in scores of quality of life.

**Characteristics**	***N* (%)**	**Quality of life mean (±SD)**
**Gender**
Male	179 (57.6)	160.66 ± 32.94
Female	132 (42.4)	165.69 ± 34.49
*p-*value		0.348
**Education level**
Primary education	63 (20.3)	164.90 ± 32.46
Secondary education	115 (37.0)	166.20 ± 35.16
Higher education	133 (42.7)	172.30 ± 32.05
*p-*value		0.223
**Marital status**
Unmarried	83 (26.7)	177.83 ± 31.68
Married	149 (47.9)	172.49 ± 30.81
Divorced	79 (25.4)	151.35 ± 33.89
*p-*value		0.000
**Residence**
City	224 (72.0)	171.27 ± 30.85
Country	87 (28.0)	161.53 ± 38.43
*p-*value		0.021
**Family yearly income, Yuan**
<50,000	124 (39.9)	162.92 ± 34.99
≥50, 000	187 (60.1)	172.28 ± 31.80
*p-*value		0.015
**Disease type**
Crohn	216 (69.5)	173.17 ± 31.78
Ulcerative colitis	95 (30.5)	158.03 ± 34.66
*p-*value		0.000
**Disease duration, years**
<2	125 (40.2)	170.24 ± 32.58
2–5	125 (40.2)	165.66 ± 34.34
>5	61 (19.6)	170.98 ± 33.07
*p-*value		0.455
**Disease activity**
Remission	209 (67.2)	178.69 ± 23.31
Active stage	102 (32.8)	147.75 ± 36.65
*p-*value		0.000

Quality of life did not differ significantly among patients of different ages, gender, education level, or disease duration groups. However, there were differences related to marital status, residence, income, disease type, and disease activity (all *P* <0.05). The details of participants' characteristics and differences in quality of life were summarized in Table 1.

### Descriptive Characteristics of Target Variables and Relationships Among Them

The mean score for perceived stigma was 71.60 ± 18.73, with 38.9% of the participants having scores above the criterion score of 80 for moderate stigma. The mean score for resilience was 95.63 ± 15.57 of 125. In terms of QOL, the mean score was 139.01 ± 21.13. The subscale of bowel symptoms had the highest mean score of the items (5.50), followed by emotional function (5.20), social function (5.16), and systemic symptoms (4.87).

Table 2 presents the results of bivariate analyses. Significant correlation was found between perceived stigma and resilience (*r* = −0.326, *P* < 0.01). All subscales of QOL were found to be negatively associated with perceived stigma but positively correlated with resilience. Moreover, patients who reported moderate or severe stigma had significantly lower scores in QOL compared with those who reported mild stigma (See detail in Table 3).

**Table 2:** Levels and association of patients' quality of life with perceived stigma and resilience (*N* = 311).

			**Correlation matrix**						
	**Mean**	**SD**	**1**	**2**	**3**	**4**	**5**	**6**	**7**
1. Perceived stigma	71.60	18.73	1						
2. Resilience	95.63	15.57	−0.326^**^	1					
3. Quality of life	139.01	21.13	−0.290^**^	0.392^**^	1				
4. Bowel symptoms	56.03	10.92	−0.198^**^	0.345^**^	0.917^**^	1			
5. Systemic symptoms	24.36	6.02	−0.173^**^	0.302^**^	0.876^**^	0.775^**^	1		
6. Emotional function	62.34	12.22	−0.354^**^	0.409^**^	0.938^**^	0.780^**^	0.773^**^	1	
7. Social function	25.82	7.51	−0.287^**^	0.330^**^	0.882^**^	0.733^**^	0.708^**^	0.790^**^	1

** *Correlation is significant at the 0.01 level (2-tailed)*.

**Table 3:** Comparison of quality of life between the patients with mild, moderate and severe perceived stigma (*N* = 311).

**Subscales of QOL**	**Mild stigma^**a**^(*N* = 190)**	**Moderate stigma^**b**^ (*N* = 115)**	**Severe stigma^**c**^ (*N* = 6)**	***P***
Bowel symptoms	57.54 ± 10.35	53.82 ± 11.56	50.50 ± 7.45	0.007
				a > b
Systemic symptoms	25.13 ± 6.00	23.26 ± 5.90	21.17 ± 4.88	0.013
				a > b
Emotional function	65.48 ± 11.30	57.99 ± 11.95	46.00 ± 6.60	0.000
				a > b,c
Social function	27.48 ± 7.04	23.49 ± 7.50	18.00 ± 6.07	0.000
				a > b,c
Total score	175.63 ± 31.44	158.56 ± 33.66	135.67 ± 18.27	0.000
				a > b,c

a,b,c *Scheffé test*.

### The Mediating Effect of Resilience on the Association Between Perceived Stigma and Quality of Life

We used “Model 4” in the PROCESS macro to test the mediating effect of resilience on the association between perceived stigma and QOL. Quality of life varied significantly depending on marital status, residence, income, disease type, and disease activity (Table 1). Therefore, those five variables were dummy coded and assigned as covariates in the test.

As seen in Table 4, the total effect of perceived stigma on all aspects of QOL were significant [bowel symptoms (β = −0.052, SE = 0.014, *P* = 0.000), systemic symptoms (β = −0.025, SE = 0.008, P = 0.002), emotional function (β = −0.109, SE = 0.016, *P* = 0.000), and social function (β = −0.052, SE = 0.010, *P* = 0.000)].

**Table 4:** Direct and indirect (mediation) effects of perceived stigma on quality of life (*N* = 311).

**Independent variable**	**Effects**	**Bowel symptoms**			**Systemic symptoms**			**Emotional function**			**Social function**		
		**Estimate**	**SE**	***P***	**Estimate**	**SE**	***P***	**Estimate**	**SE**	***P***	**Estimate**	**SE**	***P***
Perceived stigma	Total effect	−0.052	0.014	0.000	−0.025	0.008	0.002	−0.109	0.016	0.000	−0.052	0.010	0.000
	Direct effect	−0.026	0.014	0.063	−0.011	0.008	0.161	−0.076	0.016	0.000	−0.037	0.010	0.000
	Indirect effect through resilience^*^	−0.026	0.007	-	−0.014	0.004	-	−0.033	0.009	-	−0.016	0.005	-

* *The results from bias-corrected bootstrapping*.

**Figure 2:**
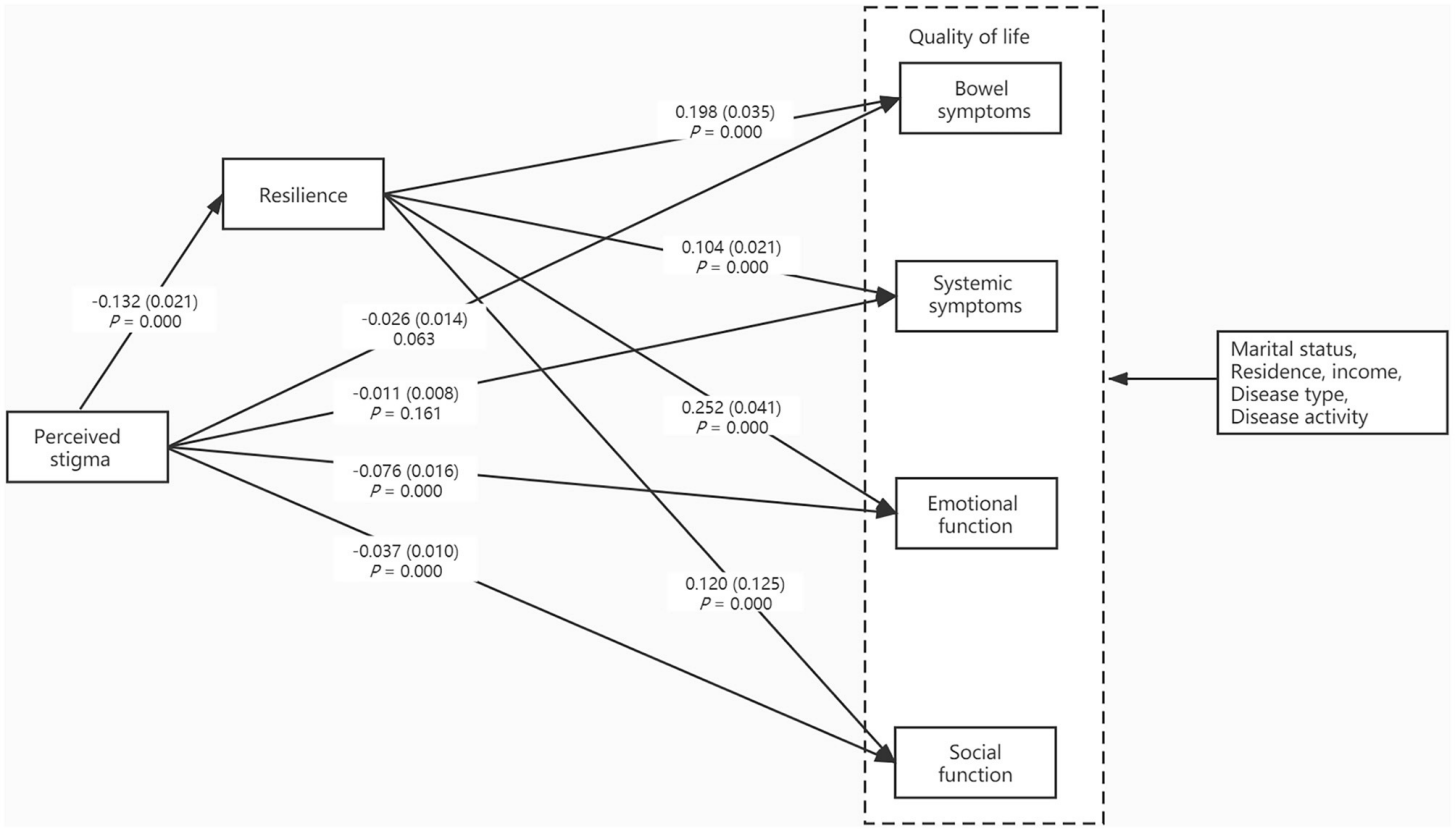
The mediation model of perceived stigma, resilience, quality of life.

Figure 2 shows the mediation model. The paths from perceived stigma through resilience to all aspects of QOL were significant. These included the products of the path from perceived stigma to resilience (*P* = 0.000) and the path from resilience to bowel symptoms (*P* = 0.000), systemic symptoms (*P* = 0.000), emotional function (*P* = 0.001) and social function (*P* = 0.000). Although perceived stigma had no direct effects on bowel symptoms and systemic symptoms, it directly affected emotional function and social function.

The result from bias-corrected bootstrapping indicated a significant indirect effect from perceived stigma to resilience to all aspects of QOL [bowel symptoms (β = −0.026, SE_Boot_ = 0.007, 95%CI: −0.041, −0.014), systemic symptoms (β = −0.014, SE_Boot_ = 0.004, 95%CI: −0.023, −0.007), emotional function (β = −0.033, SE_Boot_ = 0.009, 95%CI: −0.053, −0.018), and social function (β = −0.016, SE_Boot_ = 0.005, 95% CI: −0.026,−0.008)]. Therefore, resilience was found to mediate the relationship between perceived stigma and QOL.

In subgroup analysis stratified by disease activity, the perceived stigma was significantly negatively associated with QOL in both remission group (β = −0.304, *t* = −4.598, *P* = 0.000) and active disease group (β = −0.288, *t* = −3.005, *P* = 0.003), P_perceivedstigma−interaction_ = 0.425. Moreover, the resilience was significantly positively associated with QOL in both remission group (β = 0.401, *t* = 6.295, *P* = 0.000) and active disease group (β = 0.210, *t* = 3.811, *P* = 0.000), *P*_resilience−interaction_ = 0.639.

## Discussion

The main finding of the present study was that resilience could mediate the effect of perceived stigma on QOL in patients with IBD after statistically controlling for marital status, residence, income, disease type, and disease activity. In other words, perceived stigma might impair an individual's resilience and subsequently influenced QOL. Therefore, perceived stigma and resilience should be considered significant psychosocial variables in promoting QOL among patients with IBD.

This study revealed that the perceived stigma directly affected the emotional function and social function dimensions of QOL. Consistent with this finding, Gamwell et al. observed that more significant perceived stigma directly aggravated depressive symptoms and weakened the bonds between youth with IBD and society (45). Patients with IBD tend to perceive illness-related stigma because of public misconceptions about etiology, variability in course, and fear of incontinence, which leads to concealment and social withdrawal (22, 46). The concealment of disease could probably decrease communication and connections with others, increasing feelings of isolation and depression (47). However, since not all patients perceived stigma had a poor QOL, some authors pointed out that additional factors should be considered when explaining how psychosocial burden exerts adverse effects. Similar to earlier conclusions, we illustrated that resilience played a mediating role in the associations between perceived stigma and emotional and social function (48, 49). Resilience could help individuals adopt positive coping strategies (disclosure and seeking support) to maintain good psychosocial function (34). Higher levels of resilience are reportedly associated with fewer negative emotions and better interpersonal communication (15, 16, 50). In terms of neuro mechanisms, resilience could invoke specific brain structures and neural circuits, prompting the individual to regulate emotion and engage in prosocial behavior (17, 18).

Although we failed to find direct associations between perceived stigma and bowel symptoms and systematic symptoms dimensions of QOL, perceived stigma affected the bowel and systematic symptoms by reducing resilience. Resilience is a positive predictor of self-management in patients with chronic diseases (51, 52). Resilient patients are prone to adopt more self-management behaviors to achieve disease control (53, 54). Mediating effects between psychosocial burden and physical outcomes have been shown for various positive personality traits. Cherrington et al. reported that self-efficacy played an intermediary role in the path from depression to glycemic control in male diabetic patients (55). In a cross-sectional study conducted in patients with colorectal cancer, the variance anxiety accounted for preoperative insomnia increased after adding self-esteem to the hierarchical regression model (56). Resilience as a mediator of the association between perceived stigma and bowel and systematic symptoms has never been reported. Still, it is plausible considering that self-efficacy and self-esteem are integral parts of resilience (54). In other aspects, earlier studies demonstrated that perceived stigma was a prominent risk factor of depression and anxiety, especially for people with low resilience (49, 57, 58). The physiological mechanism of microbiota-brain-gut axis regulation could clarify how psychosocial burden can aggravate bowel and systematic symptoms in patients with IBD (59). The microbiota-brain-gut axis involved the central systems, autonomic nervous systems, endocrine system, immune system, and intestinal microenvironment. Stress responses caused by psychological disorders might activate the microbiota-brain-gut axis and further bring about altered gastrointestinal physiology, resulting in bowel and systematic symptoms (59, 60). These previous studies supported our current findings that resilience played a mediating role between perceived stigma and somatic symptoms.

Several limitations should be noted in the present study. Firstly, our study adopted a cross-sectional design, which could not confirm the causal associations between study variables. The effects of perceived stigma and resilience on QOL should be investigated and verified in more prospective studies to provide more reliable evidence. Secondly, the findings of this study should be generalized with caution because the sample in this study was mainly based on no-random participation. Random sampling could be considered in future research to confirm the findings of this study. Thirdly, except for the questionnaires measuring disease activity index, most questionnaires used in this survey, including PSS-IBD, RS-IBD, and IBDQ, came from the patients' self-reports which have subjectivities and thus easy to cause measure bias. In addition, we only included 95 patients with UC, which was insufficient to perform a subgroup analysis to determine the stability of the model in different disease types. Furthermore, the potential psychosocial mediating variables (such as self-esteem, mastery, and optimism), which are essential components of resilience, have not been analyzed. Last but not least, although CD and UC account for approximately 90% of IBD cases, there exist some patients with indeterminate colitis (1). This study only recruited patients with UC and CD. Despite the above limitations, the results presented here have significant implications. We tested the hypothesis that perceived stigma negatively affected QOL in patients with IBD and indirectly predicted QOL through resilience. The contribution of this study is to demonstrate the mediating effect of resilience on perceived stigma and QOL, which can add to the previous literature a potential mechanism whereby perceived stigma affects QOL. These findings implied that integrating intervention techniques to target resilience into the QOL improvement program of individuals with perceived stigma is feasible. There exist some resilience-enhancing programs for patients with chronic disease, such as the Be Resilient to Breast Cancer (BRBC) and Mindfulness-Based Stress Reduction (MBSR) (61, 62). Cognitive reframing is regarded as the active ingredient of resilience interventions (29). Lillis and his colleague adopted positive cognitive reframing strategies to interfere with obesity-related stigma (63). They found that the intervention group compared to the control group showed more significant mitigation of obesity-related stigma and improvement in QQL. Moreover, Kumpfer KL et al. suggested that the environmental resources (such as family and peer support) can promote resilience to help individuals positively cope with stressful events (64). A peer-led group program performed in adolescents with mental illness displayed a significant effect post-intervention, including reduced stigma stress and increased QOL (65). Therefore, resilience-oriented interventions that deliver disease knowledge and train interpersonal skills can be given to family members, peers, and coworkers of patients with IBD to increase social support and reduce perceived stigma (22, 66). As applied to clinical care, we recommend that healthcare providers incorporate perceived stigma and resilience assessment in patients' psychological screening and include cognitive reframing and support promotion in QOL improvement programs.

## Data Availability Statement

The raw data supporting the conclusions of this article will be made available by the authors, without undue reservation.

## Ethics Statement

The studies involving human participants were reviewed and approved by The Ethics Committee of the First Affiliated Hospital of Nanjing Medical University approved all study procedures (no. 2019-SRFA-122). The patients/participants provided their written informed consent to participate in this study. Written informed consent was obtained from the individual(s) for the publication of any potentially identifiable images or data included in this article.

## Author Contributions

DL and MZ designed the study, enrolled participants, analyzed and interpreted the data, and wrote the manuscript. LS, QB, ML, and SR were responsible for collecting data. ZL supervised this research project. All authors contributed to the article and approved the submitted version.

## Conflict of Interest

The authors declare that the research was conducted in the absence of any commercial or financial relationships that could be construed as a potential conflict of interest.

## Publisher's Note

All claims expressed in this article are solely those of the authors and do not necessarily represent those of their affiliated organizations, or those of the publisher, the editors and the reviewers. Any product that may be evaluated in this article, or claim that may be made by its manufacturer, is not guaranteed or endorsed by the publisher.
